# Extracellular Vesicles as Potential Biomarkers for Early Detection and Diagnosis of Pancreatic Cancer

**DOI:** 10.3390/biomedicines8120581

**Published:** 2020-12-07

**Authors:** Nelson S. Yee, Sheng Zhang, Hong-Zhang He, Si-Yang Zheng

**Affiliations:** 1 Division of Hematology-Oncology, Department of Medicine, Penn State Health Milton S. Hershey Medical Center, Hershey, PA 17033, USA; 2 Next-Generation Therapies Program, Penn State Cancer Institute, Hershey, PA 17033, USA; 3 Department of Medicine, Pennsylvania State University College of Medicine, Hershey, PA 17033, USA; 4 Micro & Nano Integrated Biosystem Laboratory, Department of Biomedical Engineering, Carnegie Mellon University, Pittsburgh, PA 15213, USA; shengzha@andrew.cmu.edu; 5 Captis Diagnostics, Inc., Pittsburgh, PA 15213, USA

**Keywords:** extracellular vesicles, exosomes, liquid biopsy, pancreatic carcinoma, early detection

## Abstract

Pancreatic carcinoma (PC) is highly metastatic, and it tends to be detected at advanced stages. Identifying and developing biomarkers for early detection of PC is crucial for a potentially curative treatment. Extracellular vesicles (EVs) are bilayer lipid membrane-structured nanovesicles found in various human bodily fluids, and they play important roles in tumor biogenesis and metastasis. Cancer-derived EVs are enriched with DNA, RNA, protein, and lipid, and they have emerged as attractive diagnostic biomarkers for early detection of PC. In this article, we provided an overview of the cell biology of EVs and their isolation and analysis, and their roles in cancer pathogenesis and progression. Multiplatform analyses of plasma-based exosomes for genomic DNA, micro RNA, mRNA, circular RNA, and protein for diagnosis of PC were critically reviewed. Numerous lines of evidence demonstrate that liquid biopsy with analysis of EV-based biomarkers has variable performance for diagnosis of PC. Future investigation is indicated to optimize the methodology for isolating and analyzing EVs and to identify the combination of EV-based biomarkers and other clinical datasets, with the goal of improving the predictive value, sensitivity, and specificity of screening tests for early detection and diagnosis of PC.

## 1. Introduction

As the seventh leading cause of cancer-related death globally, pancreatic carcinoma (PC) has remained one of the most lethal malignancies, with a 5-year relative survival rate of 9% by all stages at diagnosis [1,2]. Patients who develop PC tend to present non-specific symptoms such as anorexia, weight loss, early satiety, and abdominal or back pain. Typically, the tumor is usually diagnosed at a relatively advanced stage, such that palliative treatment and supportive care are the only options. A number of risk factors associated with PC have been identified; genetic syndromes and the underlying mutations that predispose individuals to development of PC have been revealed [3,4,5]. Any intervention to prevent PC by modifying its etiological risk factors is the key to reduction of its incidence and morality. Until effective preventive interventions are developed, early detection of PC is crucial for treatment with curative intent, typically by surgical resection of the tumor in combination with neoadjuvant and adjuvant chemotherapy and radiation therapy [4].

Conventional modalities for diagnosis of PC include imaging studies using computed tomography (CT), endoscopic retrograde cholangiopancreatography (ERCP), and upper endoscopic ultrasonography (EUS) [4]. Magnetic resonance imaging (MRI) and positron emission tomography (PET) may be indicated. Tissue diagnosis can be established by ERCP-guided brushing of bile duct or pancreatic duct with cytology, or EUS-guided fine needle aspiration of pancreatic mass. Alternatively, tissue can be obtained by CT-guided biopsy of pancreatic mass or any metastatic site using core needle or fine needle. However, these diagnostic modalities are performed following clinical presentation of suspicious signs or symptoms, and diagnosis of the tumor is relatively late in the disease course.

Serum carbohydrate antigen 19-9 (CA 19-9) has been clinically used in the diagnosis of PC, monitoring tumor response to treatment and disease progression, and detecting tumor recurrence following surgical resection [6,7,8]. However, there are limits of using serum CA 19-9 level as a screening biomarker for PC. In a study conducted in Japan, 198 of 8706 individuals with symptoms suspicious for PC (weight loss, epigastric pain, and jaundice) were found to have elevated serum levels of CA 19-9, and following evaluation, 85 patients were diagnosed with PC [9]. In a study involving 70,940 asymptomatic individuals in South Korea, the positive predictive value of elevated serum CA 19-9 for PC is 0.9% [10]. A meta-analysis of 57 studies (3285 cases of PC) and 37 studies (1882 cases of benign pancreatic disease) indicates that the combined sensitivity and specificity of CA 19-9 are 78.2% and 82.8%, respectively [11]. Considering its very low positive predictive value, together with its false positive elevation in benign hepatobiliary diseases and false negative results in 7–10% of population with the Lewis (a-/b-) genotype [12], CA 19-9 has a low utility value as a screening biomarker for PC in asymptomatic individuals.

Multiple approaches have been attempted to identify biomarkers in order to facilitate early detection of PC. Various platforms for molecular analysis of PC including genomics, transcriptomics, proteomics, and metabolomics have been developed [13]. Tumor molecular profiling has classified PC into subtypes with both diagnostic and therapeutic implications [14]. Molecular imaging using peptide ligand targeting G protein-coupled receptors [15] or cetuximab-IRDye800 [16] in PC has been reported. These molecular entities have the potential of improving detection and diagnosis of PC, and they remain to be further investigated prior to routine clinical use. Among those new technologies for early diagnosis of PC, blood-based biopsies with analysis of circulating tumor cells (CTCs), circulating tumor DNA (ctDNA), and extracellular vesicles (EVs) offer a new opportunity for early detection and diagnosis of PC [17].

As a diagnostic biomarker of PC, CTCs are associated with high specificity with a low false positive rate [18,19,20,21]. However, because CTCs are typically rare, the sensitivity of CTC-based diagnostics of PC is suboptimal particularly in early-stage disease. While ctDNA appears as a good indicator of prognosis and treatment response of PC, its sensitivity and specificity as a diagnostic biomarker of PC remains to be determined [22,23,24,25]. In recent years, the multi-faceted roles of EVs in various malignancies including PC have been under intense investigation. Molecular studies of EVs have shed new lights into the tumor biology and pathogenic mechanism of cancer [26,27,28]. Increasing evidence has indicated the promising role of EVs as a rapid minimally invasive, efficient, and cost-effective method in developing diagnostic biomarkers [29,30,31,32].

In this review, we will provide an overview of the cell biology of EVs, its isolation and analysis, and the role of EVs in various aspects of cancer biology. Particular emphasis will be focused on the roles of EVs as biomarkers for early detection and diagnosis of PC. This article is aimed to provide an updated review of EVs in PC, with the ultimate goal of developing EV-based biomarkers for effective screening and early detection of PC.

## 2. Extracellular Vesicles and Cancer Biology

EVs are extracellular membrane-enclosed vesicles released from most cell types, they are nanometer in size with diverse ranges of size from 10 to 1000 nm in diameter. EVs comprise of exosomes and microvesicles, which are also known as microparticles. Exosomes are EVs that are within the size range of 30–150 nm in diameter and derived from the multivesicular endosome pathway [33]. EVs are initially formed from intracellular compartments such as the endosomal system or shed from plasma membrane. Recent studies have shown that EVs are composed of a lipid bilayer containing transmembrane proteins and enclosing cytosolic proteins, and nucleic acids such as dsDNA, mRNA, and microRNA (miRNA) [26]. EVs play a critical role in cell-to-cell communication in neighboring cells, distant cells, and their microenvironment under both normal and pathological conditions [26]. Interestingly, EVs have been found in various fluids such as cell culture supernatant, blood, plasma, serum, urine, bile, breast milk, saliva, synovial, lacrimal, seminal, ascites, and bronchoalveolar lavage fluids, and feces.

Considering the critical messenger role of EVs in the (patho) physiological processes, new isolation methods and analytical platforms of EVs are actively being developed to address the roles of EV in disease development and progression [34]. Various methods for isolation of EVs have been developed based on the density, affinity, and size of EVs. These include ultracentrifugation, gradient ultracentrifugation, polymer coprecipitation [35], size-exclusion chromatography [36], field flow fractionation [37], microfluidic chip [38,39], contact-free sorting [40], immunoaffinity enrichment [41], and lipid nanoprobes [42,43]. Currently, ultracentrifugation is the most commonly used method for isolation of EVs. Meanwhile, various methods for molecular analysis of EVs have been developed to characterize protein and nucleic acids within EVs. These include Western blotting, ELISA, mass spectrometry, small particle flow cytometry [44], micronuclear magnetic resonance [45], nanoplasmonic exosome (nPLEX) sensor [46], integrated magnetic-electrochemical exosome (iMEX) sensor [47], electrokinetic chips [48], and ExoScreen [49]. Besides, next-generation sequencing, Sanger sequencing, droplet digital PCR, and quantitative PCR have been employed for analysis of nucleic acids within EVs. The advantage and disadvantage of those isolation methods have been detailed in other recent reviews [33,50,51,52,53,54,55,56] and summarized in Table 1.

**Figure 1:**
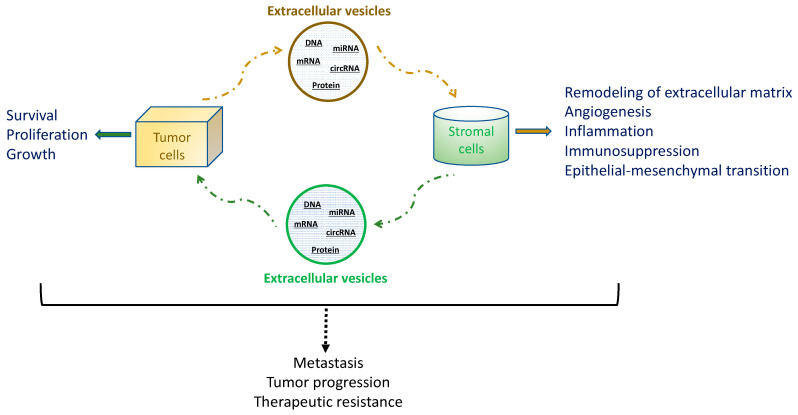
Roles of EVs in intercellular communication between tumor cells and stromal cells in local and distant microenvironments, leading to tumor metastasis, progression, and resistance to treatment.

Tumor-derived EVs are emerging as critical cell-to-cell mediators in promoting not only survival and growth of the primary tumor but also its progression and metastasis. They play an important role in intercellular communication between tumor cells and stromal cells in local and distant microenvironments [57]. Tumor-derived EVs have been shown to promote release of cancer cells into the circulation and their distant spread through communication with neighboring non-tumor cells and exerting complex effects in the primary tumor microenvironment. These include EVs-induced remodeling of the extracellular matrix, promotion of angiogenesis and inflammation, immunosuppression and protection of tumor, and epithelial–mesenchymal transition. The circulating tumor-secreted EVs are delivered to the target organ, in which tumor EVs induce the recipient cells to express cytokines that mediate activation and remodeling of stromal cells and recruitment of bone marrow-derived cells to the premetastatic niche. Ultimately, these EVs-mediated multistep processes lead to tumor progression and metastasis (Figure 1).

By facilitating the dissemination of cancer cells, EVs have been associated with progression of solid tumors, such as colon cancer, cholangiocarcinoma, hepatocellular carcinoma, pancreatic cancer, and lung cancer [58]. For instance, in an experimental model using intrasplenic injection of PAN02 murine PC cells, tumor-derived exosomes induce formation of premetastatic niche, leading to hepatic metastasis [59]. In this mouse model, PAN02-secreted exosomes expressing macrophage migration inhibitory factor are taken up by Kupffer cells in the liver. The exosomes-primed Kupffer cells release transforming growth factor-β, which induces expression of fibronectin by hepatic stellate cells. Bone marrow-derived macrophages and granulocytes are recruited to the fibronectin-enriched hepatic sites, leading to establishment of a premetastatic niche and ultimately tumor metastasis in the liver. In gastric cancer, tumor-derived exosomes promote hepatic and peritoneal metastasis by developing a liver-like microenvironment and by disrupting the mesothelial barrier, respectively [60,61]. In breast cancer cells, exosomal (mi)RNA released by cancer-associated fibroblasts enhance tumor growth, metastasis, and therapeutic resistance [62,63]; whereas cancer cells-derived exosomal miR-105 promotes tumor growth through MYC proto-oncogene (MYC)-dependent metabolic reprogramming of stromal cells [64].

Although the full spectrum of EVs biology in cancer has not been elucidated, increasing evidence supports that EVs secreted from cancer cells are important in facilitating cancer progression and metastasis. By investigating the cargos in EVs, studies show that EVs isolated from patients with cancer have the potential to serve as important diagnostic and prognostic markers [30,65,66].

## 3. Extracellular Vesicles for Early Detection and Diagnosis of Pancreatic Cancer

Bodily fluids such as plasma and serum of cancer patients may contain EVs derived from cancer and cancer-related cells. The cargo contents of EVs isolated from patients with PC can provide tumor-specific information. These include genomic DNA, mRNA, miRNA, and protein. Studies for analysis of EVs have demonstrated that some of those molecules within EVs are uniquely present or expressed in pancreatic cancer cells. Indeed, EVs have been exploited as diagnostic tools of PC.

### 3.1. EV DNA and Genomics

Detection of genetic mutations in the exosomes released from pancreatic cancer cells suggests the potential of developing exosomal DNA as diagnostic biomarkers of PC (Table 2).

Initial work has revealed the presence of single-stranded DNA such as amplification of the oncogene c-Myc in exosomes derived from the culture medium of glioblastoma cell lines [72]. Further investigation has demonstrated that exosomes contain double-stranded DNA [67], and this finding was confirmed [73]. In these studies, exosomes were isolated from pancreatic cancer cell lines and from sera in patients with PC by ultracentrifugation and treated with DNase to eliminate DNA outside exosomes. DNA was then extracted from the exosomes using the commercially available DNeasy blood and tissue kits. The isolated exosomes were found to contain >10-kilobase pair fragments of double-stranded DNA. Using Sanger sequencing, serum-derived exosomes were found to contain mutated KRAS and TP53 DNA, both of which are the most frequently mutated genes in PC [74]. Additionally, whole genome sequencing of exosomal DNA isolated from the blood of patients with PC shows that exosomes carries double-stranded DNA spanning all chromosomes.

In light of these findings, the potential clinical utility of exosomal DNA for identification of KRAS^G12D^ and TP53^R273H^ mutations was investigated in patients with pancreas-associated pathologies and in healthy human subjects [70]. Exosomes from the sera of 114 healthy subjects and 48 patients were enriched by using sequential size exclusion filtration and ultracentrifugation. Droplet digital PCR (ddPCR) analyses showed that exosomal DNA harbors KRAS^G12D^ mutation in 39.6% of cases, and TP53^R273H^ mutation in 4.2% of cases of patients with PC, while 2.6% of healthy subjects presented with KRAS^G12D^ mutation and none with TP53^R273H^ mutation in the exosomes. The highest mutation allele abundance observed was 47.45% and 0.25% for KRAS^G12D^ and TP53^R273H^ mutations, respectively.

In another study, the clinical utility of mutant KRAS at codons 12 and 13 in exosome-derived DNA from patients with early-stage PC was investigated [69]. A cumulative series of 263 individuals were studied, including a discovery cohort of 142 individuals (68 patients with PC at all stages; 20 patients with PC initially staged as localized disease and blood collected after resection for curative intent; and 54 healthy controls) and a validation cohort of 121 individuals (39 cancer patients and 82 healthy controls). The exosomes were isolated from the plasma of patients and healthy controls using serial ultracentrifugation, and the extracted DNA was then analyzed by ddPCR. Exosomal KRAS mutations were detected in 66.7% (22/33), 80% (12/15), and 85% (17/20) of localized, locally advanced, and metastatic PC, respectively, and in 7.4% (4/54) of healthy controls in the discovery cohort. In the validation cohort, mutant KRAS DNA in exosome was detected in 43.6% of early-stage PC patients and 20% of healthy controls. Furthermore, the study also demonstrated that a higher KRAS mutant allele fraction in exosome is associated with reduced disease-free survival in patients with localized disease.

In a longitudinal analysis of 34 patients with potentially resectable PC, KRAS mutant allele frequency (MAF) ≥5% in exosomal DNA significantly predicted reduced progression-free survival and overall survival [75]. Moreover, there was a significant association between a KRAS MAF >1% in exosomal DNA and tumor progression on radiographic images. Recently, a one-step EVs isolation platform has been developed by utilizing EV-matched silica nanostructures and surface conjugated lipid nanoprobe with an integrated microfluidic mixer [43]. This device was demonstrated to capture EVs from the plasma of patients with PC with an efficiency of 28.8%, and the EVs being enriched are sufficient for identification of KRAS^G12D^ or KRAS^G12V^ point mutations detected by ddPCR. Thus, this platform could have strong potential for detecting genetic mutations in diagnosis and monitoring treatment of PC.

Comprehensive profiling of genomics and transcriptomics of exosomes from patients with PC is essential to obtain useful molecular information for cancer diagnosis through liquid biopsy. Next generation sequencing (NGS) techniques including whole genome, exome, and transcriptome sequencing have been attempted as a feasibility study for comprehensive profiling of exosomal DNA and RNA [68]. The exosomes were isolated from pleural fluid, blood, and plasma from three patients with pancreaticobiliary cancers, respectively, using serial ultracentrifugation. A variety of cancer-derived biomarkers, including genetic mutations, insertions, deletions, copy number variations, and gene fusions that can act as neoantigens was detected. Potentially targetable mutations, including BRCA2 and NOTCH1, were identified. With the accumulation of exosomal profiling data, 575 protein-coding genes, 26 RNA genes and one pseudogene were also identified via data mining as directly associated to PC [76]. This exosomal database established by pure bioinformatics approaches is likely to provide a starting point for further discovery and validation of new diagnostic panels.

Results of these studies demonstrate the value of exosomal DNA as potential biomarkers for diagnosis of PC. Since healthy individuals (with no known cancer) also exhibit such mutations in their plasma EV-associated DNA, this approach may also predict the propensity towards development of PC. However, precaution should be taken that detection of a genetic mutation does not necessarily indicate any clinical evidence of cancer.

In addition, epigenetic mechanisms in cancer such as DNA methylation are also undergoing extensive research, and epigenetic alteration of oncogenes or tumor suppressor genes plays an important role in the initiation and progression of many tumors [77]. EVs are implicated in the epigenetic regulation by carrying mRNAs and proteins such as DNA methyltransferases that are involved in the process [78,79]. Yamamoto and his colleagues observed concordant methylation levels of tumor-related SOX17 gene in gastric-juice derived exosomal and nuclear DNA, suggesting that methylated DNA is efficiently packaged in exosomes [80]. Briefly after that, the group proposed that BARHL2 methylation in gastric-juice derived exoDNA could be useful for early diagnosis of gastric cancer, which yields an area under the curve (AUC) of 0.923 with 90% sensitivity and 100% specificity [81]. We anticipated that methylated exosomal DNA biomarkers dedicated to the early detection of PC will readily emerge in the near future.

### 3.2. EV miRNA and Transcriptomics

Complementary to analysis of genomic DNA, studies of exosomal miRNA and transcriptomic profiles have revealed potential biomarkers for diagnosis of PC (Table 3).

Serum exosomal miRNA levels in patients with PC and their relationships with the clinicopathologic features and prognosis have been investigated [82]. Serum exosomal miR-17-5p level was found to be higher in patients with PC than in non–PC patients and healthy participants. High levels of miR-17-5p significantly correlate with tumor metastasis and advanced stages of PC. The serum exosomal miR-21 level in PC was higher than that in the healthy and chronic pancreatitis groups, but was not significantly correlated with PC differentiation and tumor stage.

Besides, exosomal miRNAs have been shown to be potential biomarkers of PC in various settings. Analysis of a combination of proteins (CD44v6, Tspan8, EpCAM, MET, and CD104) and miRNAs (miR-1246, miR-4644, miR-3976, and miR-4306) in serum-derived exosomes improves the diagnostic accuracy of PC with a sensitivity of 1.00 (CI: 0.95–1) and specificity of 0.80 (CI: 0.67–0.90) [83]. The combination of miR-1246 and miR-4644 in salivary exosomes could be candidate biomarkers for diagnosis of pancreatobiliary tract cancer with an area under curve (AUC) of 0.833 by receiver operating characteristic (ROC) analysis [84]. In another study, miR-196a and miR-1246, are highly enriched in plasma-derived exosomes from patients with PC, and also significantly elevated in patients with PC as compared to healthy subjects [89]. Moreover, plasma exosomal miR-196a is a better indicator of PC, whereas plasma exosomal miR-1246 is good biomarker of intraductal papillary mucinous neoplasms (IPMNs) [89]. In a study of 83 patients, expression of exosomal miR-191, miR-21, and miR-451a is upregulated in in PC (*n* = 32) and IPMN (*n* = 29) as compared to controls without neoplasm (*n* = 22) [90]. Results of this study demonstrated that these exosomal miRNAs could serve as diagnostic and prognostic indicators.

Moreover, Li et al. detected a high level of miR-222 in the exosomes of a PC cell line Hs 766T through microarray analysis and then confirmed by quantitative reverse-transcription PCR (RT-qPCR) that miR-222 was significantly overexpressed in six PC cells lines in contrast to two normal pancreatic cell lines. By analyzing plasma samples of 73 patients, they further unraveled that the exosomal miR-222 level was associated with tumor size and stage [86]. Knowing that there is upregulated miR-10b expression in pancreatic cancer cells in PC [97], Korc and his colleagues developed a localized surface plasmon resonance based RNA sensing approach and showed that miR-10b levels in plasma and exosomes from normal controls and patients with chronic pancreatitis (CP) were 50- to 60-fold lower and 4- to 10-fold lower, respectively, than those in the PC samples [87]. The group later expanded the diagnostic exosomal miRNA signature to miR-10b, miR-21, miR-30c, miR-181a (overexpressed in PC), and miR-let7a (underexpressed in PC), all of which rendered an AUC of 1.0 in differentiating 29 PC patients from 11 CP patients and 6 controls [88]. So far, these studies have demonstrated the power of analyzing exosomal miRNAs either alone or in combination with exosomal protein as potential biomarkers for diagnosis of PC and for distinguishing PC from non-malignant diseases in the pancreas.

With a mouse model, Lau et al. identified seven PC-specific salivary transcriptomic biomarkers (Apbb1ip, Aspn, BCO31781, Daf2, Foxp1, Gng2, and Incenp) through a discovery-validation (microarray-qPCR) procedure [93]. They finally determined that five out of the seven mRNA, i.e. Apbb1ip, Aspn, Incenp, Daf2, and Foxp1, were significantly upregulated (*p* < 0.05) in saliva-derived exosomes of tumor-bearing mice. In a recent study, Yu et al. performed a genome-wide analysis of EV long RNA (exLR) on plasma samples of PC patients [94]. Protected from RNase in the biofluid, long RNA species including mRNA, circular RNA, and long noncoding RNA are enriched and stabilized in EVs [98,99]. To select differentially expressed exLR markers, they applied machine learning algorithms to exLR sequencing data of the samples and public RNA sequencing data sets. A support vector machine (SVM) model based on the selected panel, which comprised FGA, KRT19, HIST1H2BK, ITIH2, MARCH2, CLDN1, MAL2, and TIMP1, was constructed for diagnostic prediction of PC and it was able to identify stage I/II patients with an AUC of 0.949 in a cohort of 284 PDAC patients, 100 CP patients, and 117 healthy subjects [94]. In another study, a biochip tethered with cationic lipid–polymer hybrid nanoparticles (LPHNs) was reported that could form nanoparticle–EVs complex in 10 μL serum without EV isolation [92]. Glypican-1 mRNA was detected by amplifying fluorescence signals through catalyzed hairpin DNA circuit (CHDC) in the LPHNs. This LPHN–CHDC biochip could identify glypican-1 mRNA in serum EVs with an AUC of 1.0 or 100% specificity and 100% sensitivity in each stage of PC. Thus, the biochip has great potential to distinguish patients with early- and late-stage PC from healthy donors and patients with benign pancreatic disease.

Moreover, it is worth noting that circular RNA (circRNA) has been recently recognized as a novel class of highly stable noncoding RNA species that is abundant in exosomes [59] and may act as an exosome based circulating biomarker for early diagnosis and prognostic prediction of cancer [59,95,96]. CircRNAs can function by binding to miRNAs as sponges and suppress miRNA activity [100]. More than 1000 circRNAs are present in human serum exosomes and they are enriched compared to the parental cells [59]. Studies revealed an elevated expression level of circPDE8A [96] and circIARS [95], measured by RT-qPCR, in the tumor tissues and plasma exosomes of patients with PC. Overexpression of the exosomal circRNAs was speculated to contribute to tumor invasion and metastasis [95,96].

Combined analysis of EV miRNA, EV mRNA, and serum DNA and protein have recently been shown to improve diagnostic accuracy in PC. In a study using plasma-derived EVs from 89 patients with PC, 44 with non-cancerous pancreatic disease, and 71 healthy subjects, EVs were isolated from the subjects’ plasma using a magnetic nanopore device [91]. The tumor-derived EV miRNA and RNA, circulating cell-free DNA (ccfDNA) KRAS^G12D/V/R^ mutations, and serum CA19-9 were analyzed by application of the machine learning model. By analysis of a panel of five biomarkers including EV-miR.409, EV-CK18 mRNA, EV-CD63 mRNA, ccfDNA concentration, and CA 19-9, the algorithm-based model enables identification of patients with PC versus those without PC, with an AUC of 0.95 and accuracy of 92%, as compared to CA 19-9 (89%). Using a group of 25 patients who were considered as having no tumor metastasis based on clinical imaging and 9 of them subsequently found to have occult metastasis, the algorithm-based model achieves an accuracy of 84% for tumor staging, as compared to 64% by imaging alone.

Though the numbers of subjects in these studies are relatively small, those results provide supporting evidence that combined analysis of plasma-based exosomal miRNA/mRNA and other blood-based biomarkers improves the accuracy for diagnosis of PC. Future clinical investigations with large cohorts of patients are indicated to validate these findings.

### 3.3. EV Protein and Proteomics

Besides genomic DNA and RNA, an analysis of the EV protein and proteomic profiles have been shown to be powerful tools for early detection of PC (Table 4).

The proteoglycan glypican-1 (GPC1) in exosomes is expressed in the sera of patients with PC at very early stages but not in benign pancreatic disease [101]. GPC1 is a membrane-anchored protein that has diverse functions and it is overexpressed in several types of tumors including glioma [111], breast cancer [112], and PC [113]. It is also emerged as novel diagnostic and immunotherapeutic target in prostate cancer [114,115]. In this study, 251 patients with PC were recruited from two different cohorts (discovery and validation group). Exosomes were isolated from serum samples by ultracentrifugation, then attached to 4-μm aldehyde/sulphate latex and immunostained with anti-GPC1, and finally detected using flow cytometry. Exosomes from patients with PC expressed higher levels of GPC1 than healthy subjects. By investigating GPC1^+^ exosome in the serum of patients with PC at pre- and post-surgery stages, this study demonstrated that the percentage of GPC1^+^ exosomes increased proportionally with tumor size and correlated with the tumor burden. This finding was also confirmed by another study that investigated the GPC1 level in plasma-derived exosomes from patients with benign pancreatic disease (*n* = 16) and PC (*n* = 27) prior to pancreatectomy [104].

Additionally, KRAS codon 12 mutations detected from GPC1^+^ exosomes isolated from the plasma of patients with PC revealed identical mutation by quantitative PCR of exosomal mRNA in exosomes. Wild-type KRAS mRNA was found both in GPC1^+^ and GPC1¯ exosomes, while mutant KRAS transcript was only detected in the GPC1^+^ exosomes. Encouragingly, an AUC of 1.0 exhibiting a sensitivity and specificity of 100%, with a positive and negative predictive value of 100%, was obtained by comparing stage I–IV PC patients to healthy donors and patients with benign pancreatic diseases using GPC1^+^ exosome as a biomarker. Overall, this study provided strong evidence of exosomal GPC1 in the circulation as a good candidate for detection and screening of patients in whom PC is suspected. Two anti-GPC antibodies (ThermoFisher PA5-28055 and Sigma SAB2700282) were compared for the specific detection of GPC1^+^ plasma-derived exosomes in patients with PC as compared to healthy donors and benign pancreatic diseases using flow cytometry. Both antibodies performed well if exosomes were enriched by ultracentrifugation. The accuracy of detection was lost when exosomes were collected using isolation kits rather than ultracentrifugation, and this finding highlights the importance of the methods for isolation of exosomes [102].

Lennon et al. applied quantitative single molecular localization microscopy to assessment of exosomal EGFR and CA 19-9, two known PC-relevant membrane protein markers. Compared to healthy subjects (*n* = 6), PC patients (*n* = 5) exhibited an average of 5- and 15-fold increase in numbers of EGRF- and CA19-1 enriched EVs in plasma, respectively [109]. Lee and his colleagues, following their previous observation that alkaline phosphatase placental-like 2 (ALPPL2) was ectopically expressed in many PC cell lines at both mRNA and protein levels [116], further disclosed that the presence of ALPPL2 in PC cell-derived exosomes was quantitatively consistent with its cellular level [110]. In a study of PC induced liver premetastatic niche formation, the upregulation of the macrophage migration inhibitory factor (MIF) was found to be an early event of cancer progression that could be detected from plasma exosomes of stage I PC patients who later developed liver metastasis [59].

An analysis of the surface exosomal proteins by Castillo et al. produced a PC-specific marker panel of CLDN4, EPCAM, CD151, LGALS3BP, HIST2H2BE, and HIST2H2BF [108]. Out of a total of 7086 surface and cargo exosomal proteins in thirteen human PC cell lines and two non-neoplastic cell lines profiled by liquid chromatography–mass spectrometry (LC–MS), the candidates were selected by requiring expression in at least three PDAC cell lines with no more than one spectral count in control cell lines. The panel was used to enrich PC derived exosomes from plasma samples in an immunocapture pulldown assay and ddPCR rendered a higher detection rate of KRAS mutation in the captured exosomes than in total exosomes. Albeit this panel was not evaluated as a standalone diagnostic tool, it might facilitate enrichment of exosomal cargo for downstream molecular profiling. We note that this study [108], and several others [88,103], cast doubt on the efficacy of the aforementioned GPC1 as an optimal exosomal biomarker for PC diagnosis. They reported relatively similar exosomal GPC1 levels among normal, CP, and PC samples.

Utilizing antibodies specific for tumor antigens to capture tumor-derived extracellular vesicles (tEVs) might enable ultrasensitive assay systems for detection of cancer markers on the captured tEVs. Following isolation of total EVs by ultracentrifugation, a multiparametric profiling system incorporating arrays of nanoplasmonic sensors (NPSs) was utilized for capture of tEVs and detection of their cargo components [103]. The NPS chip contains a series of nanopores (200 nm in diameter) periodically spaced in a 100 nm-thick gold film that is coated with neutravidin. The pores are charged with biotinylated tumor antigen-binding antibodies that can capture EVs expressing these antigens. Binding of EV to the pores induces a spectral red shift of light transmitted through the nanopores. The detected light shift reflects the amount of EVs captured in the antibody-charged nanopores. To screen for the tEV antigens with high sensitivity and specificity for PC, tEVs were isolated by ultracentrifugation from the plasma of patients with PC and healthy donors. A panel of four pan-cancer markers (EGFR, EPCAM, HER2, and MUC1) and three putative PC markers (GPC1, WNT2, and GRP94) were investigated individually and together in plasma-derived exosomes. The PC^EV^ signature with the panel of markers including EGFR, EPCAM, MUC1, GPC1, and WNT2 showed improved specificity and sensitivity with an accuracy of 100% in distinguishing PC patients from healthy donors in a training cohort. Further study was conducted with a prospective cohort of 43 patients, including 35 undergoing surgery for pancreatic cancer and 8 other abdominal indications. Results show that PC^EV^ signature differentiates patients with PC from those with benign conditions and controls with an accuracy of 84%, sensitivity of 86%, and specificity of 81%. These data demonstrate the PC^EV^ signature of total EVs offered higher sensitivity, specificity, and accuracy than analysis of the existing serum marker CA 19-9 or single tEVs marker.

Furthermore, Liang et al. devised an ultrasensitive nanoplasmon-enhanced scattering (nPES) assay to directly quantify tEVs from small volumes of unprocessed plasma [107]. EVs were first captured by EV-specific antibodies on the surface of a sensor chip and then bound to antibody-conjugated gold nanospheres and nanorods. The group identified ephrine type-A receptor 2 (EphA2) as a candidate EV biomarker that was significantly overexpressed in PC cell lines using LC–MS based proteomics and bioinformatics approach, and demonstrated its effectiveness in distinguishing early stage PC patients from CP patients with an AUC of 0.94 and from healthy individuals with an AUC of 0.96. In another study, Kenwarr et al. presented a microfluidic chip that is fabricated in polydimethylsiloxane and functionalized with antibodies against CD63 and employed the device to isolate and quantify exosomes from serum samples [85]. As a proof of concept, characterization of the chip-bound exosomes was performed through Western blot and miRNA openarray analysis. Alteration in the expression level in isolated exosomes of patients with PC was reported for proteins CD63 (3.17 fold) and Rab5 (1.73 fold), and some miRNAs (>1.5 fold-change for hsa-miR-130a, hsa-miR-29b, hsa-miR-30b, hsa-miR-518d, hsa-miR-551b, and hsa-miR-646, and <0.67 fold-change for hsa-miR-601, hsa-miR-106b, hsa-miR-92a, hsa-miR-1275, and hsa-miR-302c*). The diagnostic power of these molecules remains to be established in further studies.

Protein identified from proteomic analysis of exosomes derived from the pancreatic cancer cell line has been demonstrated to have potential of a diagnostic biomarker of PC. The zinc transporter protein ZIP4 was found to be an upregulated exosomal protein that is growth-promoting in a pancreatic cell line. In a study using serum-derived EVs from 24 patients with PC and 32 patients with non-malignant pancreatic diseases, the elevated serum levels of exosomal ZIP4 in patients with PC showed a diagnostic value of AUC 0.89 [106]. Further study is indicated if combined analysis of exosomal ZIP4 with other blood-based biomarkers improves diagnostic accuracy for PC.

An integrative method to capture EVs from blood, plasma, or serum onto an alternating current electrokinetic microarray chip and analyze by immunofluorescence was developed. In a validation study of 20 patients with PC and 11 healthy subjects, analysis of the exosomal protein biomarkers, glypican-1, and CD63 enabled diagnosis of PC with a sensitivity of 99% and specificity 82% [48]. This study features the speed and simplicity of this device for capturing and detection of exosomal biomarkers on the chip.

Besides exosomes derived from plasma and serum, the feasibility of isolation of exosomes from pancreatic duct fluid and examination of exosomal proteins for diagnosis of PC was investigated [105]. Exosomes were isolated by serial ultracentrifugation from pancreatic duct fluid collected from a total of 26 patients with PC, intraductal papillary mucinous neoplasm, and other benign pancreatic diseases, at resection. The proteins in the isolated exosomes were identified by mass spectrometry (MS). This study found that the top 35 proteins identified from LC–MS were able to distinguish PC from benign and preneoplastic diseases. Those identified proteins include carcinoembryonic antigen-related cell adhesion molecule 1 (CEARCAM1), CEARCAM5, tenascin C, matrix metalloproteinase-7, laminin subunit beta-3, and laminin subunit gamma-2.

Analyses of exosomal proteins have the potential to significantly enhance the sensitivity and specificity of PC diagnosis. In combination with exosomal DNA and miRNAs, those exosomal proteins extracted from the plasma or pancreatic duct fluid may prove to be clinically useful biomarkers for early detection of PC.

## 4. Conclusions and Future Perspectives

Despite advances in understanding its pathogenetic mechanism, PC has remained a great oncological challenge, and early detection is the key to reducing its disease burden and lethality. Liquid biopsy particularly plasma-based EVs with an analysis of their molecular contents have emerged as a promising diagnostic tool for early detection of PC. Multiplatform analyses of plasma-based exosomes for genomic DNA, miRNA, mRNA, and protein have demonstrated excellent sensitivity and specificity for diagnosis of PC. However, there is no Food and Drug Administration approved assay available for PC diagnostic purpose, the methodology for isolating and analyzing plasma-based EVs remains to be optimized by improving its efficiency and cost effectiveness.

**Figure 2:**
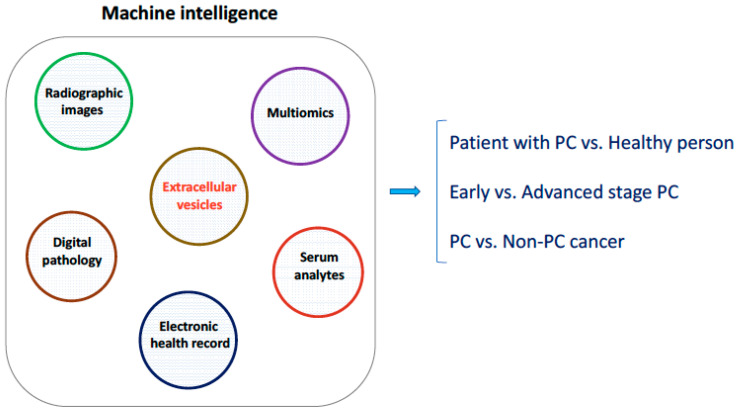
Integrative analysis of circulating EV-derived biomarkers with other datasets using machine learning-based algorithms for detection of pancreatic cancer (PC) in asymptomatic individuals, detection of PC at early stage, and distinguishing PC from other types of cancer.

For mass screening of asymptomatic individuals, circulating EV-based biomarkers with high positive predictive value and low false positive or negative value will be essential. To detect PC at its earliest stages in patients with or without symptoms, highly sensitive EV-based biomarkers specific to pancreatic cancer (as compared to other types of cancer) are indicated. Combination of EV-based biomarkers and serum analytes such as protein and miRNA is important to develop a molecular signature for diagnosis of PC. Machine learning-based algorithms for integrative analysis of circulating EV-derived biomarkers along with digitized images, multiomics, clinical datasets, and population health are expected to advance detection of PC (Figure 2). Affordable cost, efficient testing, and validation in large multisites clinical studies will be necessary for development of effective screening modality that utilizes EV-based biomarkers for early detection and diagnosis of PC.

## Figures and Tables

**Table 1:** Comparison of existing techniques for isolation and enrichment of extracellular vesicles (EVs).

Enrichment Technique	Mechanism	Speed/Rate	Volume	Efficiency	Purity	Cost	Point of Care
Ultracentrifugation	Density	2–24 h	0.5–5 mL	2–25%	Medium/High	$$$	No
Immunocapture	Antigen	1–5 h	500 µL	N/A	Medium/High	$$	Somewhat
Precipitation	Solubility	12–24 h	1–10 mL	N/A	Low	$	No
Lipid Nanoprobe	Affinity	15 m	up to 1 mL	50–80%	Medium	$$	Somewhat
LNP Nanostructure Platform	Affinity & Size	2 m–3 h 20 m	up to 2 mL	30%	High	$	Yes
	Poor		Acceptable			Good	

$$$: high cost; $$: medium cost; $: low cost. This table is modified from Wan et al. 2019 (Lab Chip 19: 2346–2355; https://doi.org/10.1039/C8LC01359D) with permission for use of this table from The Royal Society of Chemistry.

**Table 2:** Panels of EV DNA biomarkers for early detection and diagnosis of pancreatic carcinoma.

Classification	Biomarkers	Source	Method for Isolation of EVs	Method for Analysis of Biomarkers	Diagnostic Performance	Reference
**DNA mutation**	*KRAS, TP53*	Serum	Ultracentrifugation	Sanger sequencing	ND	Kahlert et al. [67]
	*NOTCH1*, *BRCA2*	Pleural fluid, blood, plasma	Ultracentrifugation	NGS	ND	San Lucas et al. [68]
	*KRAS*	Plasma	Ultracentrifugation	ddPCR	ND	Allenson et al. [69]
	*KRAS*, *TP53*	Serum	Size exclusion filtration, ultracentrifugation	ddPCR	ND	Yang et al. [70]
	*KRAS*	Plasma	Ultracentrifugation	ddPCR	ND	Bernard et al. [71]
	*KRAS*	Plasma	Lipid nanoprobe	ddPCR	Capture efficiency 28.8%	Wan et al. [43]

AUC: area under curve; ddPCR: digital droplet polymerase chain reaction; EV: extracellular vesicles; ND: not determined; NGS: next-generation sequencing.

**Table 3:** Panels of EV RNA biomarkers for early detection and diagnosis of pancreatic carcinoma.

Classification	Biomarkers	Source	Method for Isolation of EVs	Method for Analysis of Biomarkers	Diagnostic Performance	Reference
**miRNA**	miR-17-5p, miR-21	Serum	Ultracentrifugation	RT-PCR	ND	Que et al. [82]
	miR-1246, miR-4644, miR-3976 and miR-4306, CD44v6, Tspan8, EpCAM, MET, CD104	Serum	Sucrose-gradient centrifugation	Microarray, flow cytometry	Sensitivity 1.00, Specificity 0.80	Madhavan et al. [83]
	miR-1246, miR-4644	Saliva	Total exosome isolation reagent	RT-qPCR	AUC 0.833	Machida et al. [84]
	miR-130a, miR-29b, miR-30b, miR-518d, miR-551b miR-646, miR-601, miR-106b, miR-92a, miR-1275, miR-302c*	Serum	ExoChip	miRNA openarray analysis	ND	Kanwar et al. [85]
	miR-222	Cell culture media	Total exosome isolation kit	RT-qPCR	ND	Li et al. [86]
	miR-10b	Plasma	Ultracentrifugation	Localized surface plasmon resonance-based assay	ND	Joshi et al. [87]
	miR-10b, miR-30c	Plasma	Ultracentrifugation	RT-qPCR	AUC 1.0	Lai et al. [88]
	miR-196a, miR-1246	Plasma	Ultracentrifugation	RT-qPCR	AUC 0.81	Xu et al. [89]
	miR-191, miR-21, miR-451a	Plasma	Exosome isolation kit	NGS, RT-qPCR	Sensitivity 65.8–80.7% Specificity 81–85.7% Accuracy 73.6–80.8%	Goto et al. [90]
	hsa.miR.409	Plasma	Tracked etched magnetic nanopore device	NGS, RT-qPCR	AUC 0.93	Yang et al. [91]
**mRNA**	GPC1	Serum	None	LPHN–CHDC biochip	AUC 1.0	Hu et al. [92]
	Apbb1ip, Aspn, Incenp, Daf2, Foxp1	Saliva	Ultracentrifugation	RT-qPCR	ND	Lau et al. [93]
	FGA, KRT19, HIST1H2BK, ITIH2, MARCH2, CLDN1, MAL2 and TIMP1	Plasma	exoRNeasy Serum/Plasma Kit	Long RNA sequencing	AUC 0.949	Yu et al. [94]
	CK18, CD63	Plasma	Tracked etched magnetic nanopore device	NGS, RT-qPCR	AUC 0.93	Yang et al. [91]
**circRNA**	circIARS	Plasma	Total exosome isolation kit	RT-qPCR	ND	Li et al. [95]
	circPDE8A	Plasma	Not mentioned	RT-qPCR	ND	Li et al. [96]

AUC: area under curve; ddPCR: circRNA: circular RNA; digital droplet polymerase chain reaction; EV: extracellular vesicles; LPHN–CHDC: confine catalyzed hairpin DNA circuit in cationic lipid-polymer hybrid nanoparticles; miR: microRNA; ND: not determined; NGS: next-generation sequencing; NPS: nanoplasmonic sensors; RT-qPCR: reverse-transcription quantitative polymerase chain reaction.

**Table 4:** Panels of EV protein biomarkers for early detection and diagnosis of pancreatic carcinoma.

Classification	Biomarkers	Source	Method for isolation of EVs	Method for Analysis of Biomarkers	Diagnostic Performance	Reference
**Protein**	GPC1	Serum	Ultracentrifugation	Flow cytometry	AUC 1.0	Melo et al. [101,102]
	EGFR, EPCAM, MUC1, GPC1, WNT2	Plasma	Ultracentrifugation	NPS	Accuracy 84%	Yang et al. [103]
	GPC1	Plasma	Ultracentrifugation	ELISA	AUC 0.59	Frampton et al. [104]
	Combination of 35 proteins	Pancreatic duct fluid	Ultracentrifugation	LC–MS	ND	Zheng et al. [105]
	ZIP4	Serum	Exosome isolation kit	Immunoprecipitation	AUC 0.89	Jin et al. [106]
	CD63, GPC1	Blood, plasma, serum	Alternating current electro-kinetics microarray chip	Immunofluorescence	Sensitivity 99% Specificity 82%	Lewis et al. [48]
	MIF	Plasma	Ultracentrifugation	ELISA	ND	Costa-Silva et al. [59]
	EphA2	Plasma	None	Nanoplasmon-enhanced scattering assay	AUC 0.96 (PC versus NC), AUC 0.93 (PC versus CP)	Liang et al. [107]
	CLDN4, EPCAM, CD151, LGALS3BP, HIST2H2BE, HIST2H2BF	Plasma	Ultracentrifugation	ddPCR	ND	Castillo et al. [108]
	EGFR, CA19-9	Plasma	Size exclusion chromatography	Quantitative single molecule localization microscopy	ND	Lennon et al. [109]
	ALPPL2	Cell culture media	Ultracentrifugation	Aptamer-based ELISA (ALISA)	ND	Shin et al. [110]
	CD63, Rab5	Serum	ExoChip	Western blot	ND	Kanwar et al. [85]

AUC: area under curve; ELISA: enzyme-linked immunosorbent assay; EV: extracellular vesicles; GPC1: glypican-1; LC–MS: liquid chromatography–mass spectrometry; ND: not determined.
